# Establishing a Remote Patient Monitoring Infrastructure with Starlink in Indonesia for the Aging Population to Improve Alzheimer’s and Dementia Care

**DOI:** 10.1002/alz.093132

**Published:** 2025-01-09

**Authors:** Ida Bagus Kresnasandi, Brian B Johnson

**Affiliations:** ^1^ University of Minnesota, Minneapolis, MN USA

## Abstract

**Background:**

Modernizing the remote patient monitoring (RPM) infrastructure in Indonesia has historically been limited by the unmet need of access to internet connectivity. New advancements made possible by Starlink and the internet of things (IoT) for RPM present new opportunities to connect people separated by geography and diverse cultural variations which includes over 700 languages and dialects. Alzheimer’s disease has increased by 87% from 2019 to 2022 and similar challenges in the United States apply to Indonesia; the cost of care, nurse shortage, and large aging population. Clinicians, scientists, and engineers now have the potential to solve the unmet need of insufficient aging care by leapfrogging previous technologies with the latest in artificial intelligence (AI) and digital health (DH).

**Method:**

Establishing internet connection across the 16,771 islands is the first step to creating a communication network that provides the aging population in Indonesia access to the benefits of modern healthcare. With consistent and reliable access to internet access through Starlink satellite broadband internet a constellation of devices can be utilized in urban and remote areas such as the 12,000 villages that have no 4G internet access. For example WhatsApp is currently being tested to connect patients to clinicians because this application does not need a cellular plan. A pilot study conducted by Udayana University, Bali, could test the feasibility of delivering a RPM system to rural areas such as Batur village and the island of Nusa Penida.

**Result:**

Solving the problem of lack of access to internet connectivity in Indonesia creates tremendous opportunity to deliver state of the art AI and DH tools to clinicians for Alzheimer’s, dementia care and healthcare overall, reducing health disparities on a national level.

**Conclusion:**

Exporting the latest innovations from the top biomedical research institutions in the US to Indonesia could benefit the aging population by increasing their quality of life. Starlink is a key component of establishing access to communication infrastructure by allowing Indonesia’s public health insurance system, BPJS, to reach people in remote places.

